# A human-like bile acid pool induced by deletion of hepatic *Cyp2c70* modulates effects of FXR activation in mice[S]

**DOI:** 10.1194/jlr.RA119000243

**Published:** 2019-09-10

**Authors:** Jan Freark de Boer, Esther Verkade, Niels L. Mulder, Hilde D. de Vries, Nicolette Huijkman, Martijn Koehorst, Theo Boer, Justina C. Wolters, Vincent W. Bloks, Bart van de Sluis, Folkert Kuipers

**Affiliations:** Departments of Laboratory Medicine* University Medical Center Groningen, University of Groningen, Groningen, The Netherlands; Pediatrics,† University Medical Center Groningen, University of Groningen, Groningen, The Netherlands; iPSC/CRISPR Center Groningen,§ University Medical Center Groningen, University of Groningen, Groningen, The Netherlands; University of Groningen,§ Campus Fryslân, Leeuwarden, The Netherlands

**Keywords:** liver, humanized mouse model, cholesterol, transintestinal cholesterol excretion, cytochrome P450 family 2 subfamily c polypeptide 70

## Abstract

Bile acids (BAs) facilitate intestinal absorption of lipid-soluble nutrients and modulate various metabolic pathways through the farnesoid X receptor (FXR) and Takeda G-protein-coupled receptor 5. These receptors are targets for therapy in cholestatic and metabolic diseases. However, dissimilarities in BA metabolism between humans and mice complicate translation of preclinical data. Cytochrome P450 family 2 subfamily c polypeptide 70 (CYP2C70) was recently proposed to catalyze the formation of rodent-specific muricholic acids (MCAs). With CRISPR/Cas9-mediated somatic genome editing, we generated an acute hepatic *Cyp2c70* knockout mouse model (*Cyp2c70*^ako^) to clarify the role of CYP2C70 in BA metabolism in vivo and evaluate whether its activity modulates effects of pharmacologic FXR activation on cholesterol homeostasis. In *Cyp2c70*^ako^ mice, chenodeoxycholic acid (CDCA) increased at the expense of βMCA, resulting in a more hydrophobic human-like BA pool. Tracer studies demonstrated that, in vivo, CYP2C70 catalyzes the formation of βMCA primarily by sequential 6β-hydroxylation and C7-epimerization of CDCA, generating αMCA as an intermediate metabolite. Physiologically, the humanized BA composition in *Cyp2c70*^ako^ mice blunted the stimulation of fecal cholesterol disposal in response to FXR activation compared with WT mice, predominantly due to reduced stimulation of transintestinal cholesterol excretion. Thus, deletion of hepatic *Cyp2c70* in adult mice translates into a human-like BA pool composition and impacts the response to pharmacologic FXR activation. This *Cyp2c70*^ako^ mouse model may be a useful tool for future studies of BA signaling and metabolism that informs human disease development and treatment.

Bile acids (BAs) are amphipathic steroids, produced exclusively by the liver from cholesterol, that act as soaps to facilitate solubilization and absorption of dietary cholesterol, fats, and fat-soluble vitamins in the small intestine (1). Whereas cholesterol is virtually not absorbed in the absence of BAs, fatty acid absorption decreases from ∼97% to ∼70% when no BAs are present within the intestinal lumen (2). The capacity to solubilize cholesterol, but also other lipids, in the intestine is largely determined by the size and composition of the circulating BA pool that consists of several species differing in number, position, and orientation of hydroxyl groups (**Table 1**) and hence in lipid-dissolving properties (1). BAs also act as signaling molecules by activating the nuclear farnesoid X receptor (FXR; NR1H4) (3, 4) and the membrane-bound Takeda G-protein-coupled receptor 5 (TGR5; GPBAR1) (5, 6). FXR plays a prominent role in the regulation of BA synthesis and profoundly impacts on cholesterol metabolism (7–9). Moreover, FXR affects several aspects of glucose and energy homeostasis (10–13). TGR5 also impacts on the regulation of energy metabolism by inducing the release of GLP-1 from intestinal L-cells and by stimulating brown adipose tissue activity (13–15). Importantly, the various BA species differ widely in their ability to activate FXR (3, 4) and TGR5 (5, 6). Hence, it is increasingly appreciated that size, cycling frequency, and composition of the BA pool represent important factors in maintenance of metabolic control. BA signaling pathways are therefore considered attractive therapeutic targets for treatment of metabolic syndrome-associated morbidities, including nonalcoholic fatty liver disease (16, 17).

**TABLE 1.** Hydroxylation patterns of major BA species

	Position and Configuration of BA Hydroxyl Groups			
	C3-OH	C6-OH	C7-OH	C12-OH
Monohydroxylated				
Lithocholic acid	α	—	—	—
Dihydroxylated				
Chenodeoxycholic acid	α	—	α	—
Ursodeoxycholic acid	α	—	β	—
Deoxycholic acid	α	—	—	α
Hyodeoxycholic acid	α	α	—	—
Murideoxycholic acid	α	β	—	—
Trihydroxylated				
Cholic acid	α	—	α	α
αMuricholic acid	α	β	α	—
βMuricholic acid	α	β	β	—
ωMuricholic acid	α	α	β	—
Hyocholic acid	α	α	α	—

Current knowledge concerning the impact of BAs and their signaling pathways on metabolism has largely been derived from studies in mice. Mouse models are also often used for initial testing of potential novel therapeutics. However, physiologically very relevant differences exist in the composition of the circulating BA pool between humans and mice. The human liver produces two primary BAs, i.e., cholic acid (CA) and chenodeoxycholic acid (CDCA), that can be converted by the microbiome into the secondary deoxycholic acid (DCA) and lithocholic acid (LCA), respectively. Because the latter is poorly absorbed, the human BA pool generally consists of a mixture of mainly CA, DCA, and CDCA (18). The BA pool in mice is more heterogeneous. Whereas CDCA is an end product of BA synthesis in humans, it is additionally hydroxylated in the murine liver (1), generating trihydroxylated α and β muricholic acids (MCAs) that comprise ∼40% of the murine BA pool. Opposite to humans, only minute amounts of CDCA are found in mice (18). In contrast to the relatively hydrophobic CDCA molecules, MCA species are found at the hydrophilic end of the spectrum (19). Consequently, mixed micelles composed of MCAs have a lower capacity to solubilize cholesterol and other lipids compared with mixed micelles composed of more hydrophobic BA species. The ability of the rodent-specific MCAs to activate FXR and TGR5 also differs tremendously from the BA species present in humans. CDCA is the most potent endogenous FXR agonist (3, 4, 20), whereas taurine-conjugated α- and βMCA have been reported to exert antagonistic activity toward this receptor (21). The potential to activate TGR5 also differs considerably between BAs, the more hydrophobic species being the better agonists (5, 6). The entirely different properties of MCAs as compared with CDCA complicates translation of rodent data to the human situation.

Recently, the enzyme Cytochrome P450 family 2 subfamily c polypeptide 70 (CYP2C70) was proposed to catalyze the production of MCAs in mice (22). *Cyp2c70*-deficient mice have not been described so far, yet, mice lacking all 15 *Cyp2c* genes except *Cyp2c44* were shown not to produce MCAs (22). Conversely, when human HepG2 cells were transfected with the murine *Cyp2c70* gene, these cells became able to produce αMCA and βMCA when CDCA and UDCA, respectively, were added to the culture medium (22). CYP2C70 is therefore considered a 6β-hydroxylase producing αMCA from CDCA and βMCA from UDCA.

In the present study, we employed CRISPR/Cas9-mediated somatic genome editing to inactivate the gene encoding CYP2C70 in the adult mouse liver and, thereby, to generate a mouse model with a more human-like BA pool composition. Our approach resulted in drastically decreased hepatic CYP2C70 levels, an increased contribution of CDCA, and a marked reduction of βMCA but not of αMCA in the BA pool. Using in vivo administration of stable isotopically labeled BA tracers, we demonstrate that CYP2C70 catalyzes the production of βMCA by a two-step reaction involving 6β-hydroxylation of CDCA, producing αMCA, followed by epimerization of the C7 hydroxyl group from the α to the β orientation, which generates βMCA. Furthermore, the more human-like BA pool composition profoundly influenced the effects of pharmacological FXR activation on cholesterol homeostasis.

## METHODS

### Animals

Liver-specific Cas9-transgenic (L-Cas9tg) mice on a C57BL/6J background (23) were used for the experiments. In these mice, hepato-selective editing of genes in the adult livers can be accomplished by delivery of guide RNA molecules to the Cas9-expressing hepatocytes (23). An adenovirus encoding a single-guide RNA (sgRNA) targeting the murine *Cyp2c70* gene (described below) was used to acutely inactivate this gene in the liver. To ablate hepatic *Cyp2c70*, 8- to 10-week-old male and female mice were injected with 1 × 10^11^ particles of the sgRNA-encoding adenovirus. All mice were housed individually in temperature-controlled animal rooms with a 12 h light/12 h dark cycle. Female mice were used for the tracer experiments with D4-CDCA, D4-UDCA, and D5-αMCA, whereas male mice were used for all other experiments. Experiments were performed 4 weeks after virus injection. All experiments were performed in accordance with the Dutch law and were approved by the Dutch Central Committee for Animal Experiments and the Animal Welfare Body of the University of Groningen.

### Production and purification of the adenovirus

Three different sgRNAs were tested in vitro for the ability of the sgRNA-Cas9 complexes (24) to induce indels in the *Cyp2c70* gene using a Surveyor Mutation Detection kit (Integrated DNA Technologies, Leuven, Belgium). Based on the results, the sgRNA with the guide sequence 5′-CCCACTCCTTTACCAATTGT-3′ (see supplemental Fig. S1 for full sequence of the expression cassette) was selected and the promoter and sgRNA were cloned into the pENTR2B entry vector of the Virapower Adenoviral Gateway system (Thermo Fisher Scientific, Waltham, MA). The whole expression cassette was then recombined into the pAd/PL-DEST adenoviral vector using LR Clonase (Thermo Fisher Scientific). Adenovirus was produced by transfection of *Pac*I-digested vector into HEK293A cells, which contain a stably integrated copy of the adenoviral E1 gene necessary for virus replication. When cytopathic effects were present, crude viral lysate was harvested and used to infect fresh HEK293A cells for several rounds of expansion of virus production. After the final infection, cells from 50 dishes (145 cm^2^) were harvested, centrifuged at 250 *g*, and the pellet was resuspended in 10 mM Tris (pH 8.0). Next, three freeze-thaw cycles were performed and the lysate was centrifuged at 1,500 *g* for 20 min. The supernatant was then loaded on top of a cesium chloride gradient consisting of 8 ml 73.4% (w/v) and 12 ml 28.9% (w/v) solutions. The gradient was centrifuged at 100,000 *g* and 4°C for 2 h. The lower of two visible bands, representing the DNA-containing virus particles, was harvested and placed on top of a new cesium chloride gradient (see above) for further purification. This second gradient was centrifuged at 100,000 *g* and 4°C for 16 h. Again, the lower of the two visible bands was harvested for further purification using 10 DG columns (Bio-Rad, Hercules, CA). The virus concentration was then quantified by spectrophotometry at a wavelength of 260 nm and aliquots were frozen at −80°C until use. Mice were intravenously injected with 1 × 10^11^ particles in 200 μl of PBS. A virus with no guide RNA sequence was used as a control in the experiments when indicated.

### Oral and intravenous administration of labeled BAs

Animals received 0.50 mg of either D4-taurochenodeoxycholic acid or D4-tauroursodeoxycholic acid (TUDCA) (Cambridge Isotope Laboratories, Inc., Andover, MA) in PBS by oral gavage. Gallbladder cannulation (7) and bile collection were performed 16 h after administration of the tracers to these animals. Conversion of the administered BA tracers into other species was determined as described below. Separate groups of animals were intravenously infused with either D4-CDCA or D5-αMCA (Cambridge Isotope Laboratories, Inc.). Mice were anesthetized by intraperitoneal administration of Hypnorm (1 ml/kg fentanyl-fluanisone) and diazepam (10 mg/kg). A catheter was inserted into the right jugular vein, after which the common bile duct was ligated and the gallbladder was cannulated. After collection of a baseline sample (20 min), either D4-CDCA (3.25 nmol/min) or D5-αMCA (1.05 nmol/min) were infused for 2 h. Bile collected during the first 20 min (*t* = 0), from 40 to 60 min, and from 100 to 120 min was used for quantification of label enrichment in the different BA species by GC-electron capture negative ionization MS of pentafluorobenzyl ester-TMS derivatives (25), which allows for highly sensitive quantification of BA isotopomers.

### BA measurements

BAs in plasma and bile were measured by ultra (U)HPLC-MS/MS on a Nexera X2 UHPLC system (Shimadzu, Kyoto, Japan), coupled to a SCIEX QTRAP 4500 MD triple quadrupole mass spectrometer (SCIEX, Framingham, MA) and quantified using D4-labeled BAs as internal standards (26). For fecal BA measurements, feces were dried and thoroughly ground. About 50 mg were incubated for 2 h at 80°C in 1 ml of alkaline methanol and purified using C18 cartridges (Waters, Milford, MA). BAs were methylated and trimethylsilylated prior to quantification by gas-LC using 5β-cholanic acid 7α,12α diol as internal standard as described (27).

### Fecal neutral sterol measurements

Feces were dried and thoroughly ground. About 50 mg of feces were incubated for 2 h at 80°C in alkaline methanol. Neutral sterols were extracted three times with 3 ml of petroleum benzine, dried under nitrogen flow, and derivatized by adding 100 μl of pyridine/*N*,*O*-Bis(trimethylsilyl)trifluoroacetamide (BSTFA)/trimethylchlorosilane in a ratio of 50:50:1. Following incubation for 1 h at room temperature, the solution containing the derivatized neutral sterols was evaporated under nitrogen and 1 ml of heptane with 1% BSTFA was added to the dry neutral sterols for quantification of the concentrations by GC (Agilent 6890, Amstelveen, The Netherlands) using a CPSil 19 capillary column (25 m × 0.25 mm × 0.2 μm) (Chrompack, Middelburg, The Netherlands) and 5α-cholestane as an internal standard (27).

### Gene expression analysis

RNA was extracted from tissue using TRI-Reagent (Sigma, St. Louis, MO) and 1 μg was reverse transcribed using Moloney-Murine Leukemia Virus reverse transcriptase (Life Technologies, Bleiswijk, The Netherlands). Real-time quantitative PCR (qPCR) was performed on a QuantStudio-3 real-time PCR system (Applied Biosystems, Foster City, CA), using TaqMan primer-probe combinations. Data were normalized to cyclophilin as a housekeeping gene and further normalized to the mean of the respective control group.

### Histology

Tissues were rapidly excised after euthanization, fixed in formalin (4%), and embedded in paraffin. Sections of 4 μm were used for hematoxylin and eosin staining according to standard protocols. Slides were scanned using a Scanscope CS pathology scanner (Aperio Technologies, Vista, CA); images were obtained using ImageScope Viewer software (V11.2.0.780; Aperio Technologies).

### Microbiota analysis

Composition of the microbiota was analyzed by sequencing of 16S ribosomal DNA isolated from freshly frozen feces that were collected 23 days after virus injection, essentially as described previously (28). Briefly, the mice were placed on a clean surface and monitored continuously. As soon as a fecal pellet was dropped, it was picked up using a sterile forceps, put into a sterile tube, snap-frozen in liquid nitrogen, and stored at −80°C until analysis. Bacterial DNA was isolated and hypervariable regions of 16S DNA were sequenced by Novogene (Wan Chai, Hong Kong). DNA amplification was performed on an Illumina HiSeq platform to generate 250 bp paired-end reads. Species diversity within samples was analyzed by clustering all effective tags to operational taxonomic units (OTUs) at 97% similarity. Species were annotated based on the OTUs’ representative tags.

### Measurement of proteins in liver

Protein levels of CYP2C70, CYP7A1, CYP8B1, and CYP27A1 were quantified from liver tissue homogenates using targeted proteomics (7). Briefly, in-gel digestion was performed on 50 μg of total protein using trypsin (1:100 g/g sequencing grade modified trypsin V5111; Promega). After reduction with 10 mmol/l of dithiothreitol and alkylation with 55 mmol/l of iodoacetamide, solid-phase extraction [SPE C18-Aq (50 mg/1 ml); Gracepure; Thermo Fisher Scientific] was performed for sample clean-up.

LC on a nano-UHPLC system (Ultimate UHPLC focused; Dionex; Thermo Fisher Scientific) was performed to separate the peptides. The target peptides were analyzed by a triple quadrupole mass spectrometer equipped with a nano-electrospray ion source (TSQ Vantage; Thermo Fisher Scientific). For the LC-MS measurements, an amount of the digested peptides equivalent to 1 μg total protein starting material was injected together with 50 fmol of stable isotopically labeled standard peptide for CYP2C70 (TDSSLLSR, PEPotec grade 2; Thermo Fischer Scientific) and 4 fmol of isotopically labeled concatemer-derived standard peptides for CYP7A1 (LSSASLNIR), CYP8B1 (VVQEDYVLK), and CYP27A1 (LYPVVPTNSR) (QconCAT technology, PolyQuant GmbH, Germany). The endogenous peptide concentrations were quantified using the known concentration of the isotopically labeled standard peptides.

### Hepatic lipid measurements

Liver lipids were extracted from homogenates according to Bligh and Dyer (29). Cholesterol and triglyceride concentrations in the extracts were measured using commercially available reagents (DiaSys Diagnostic Systems and Roche Diagnostics), whereas phospholipids in liver extracts were quantified as described (30). All hepatic lipids are expressed as micromoles per gram of liver.

### Plasma parameters

Plasma triglycerides, free fatty acids, total cholesterol, and free cholesterol were measured using commercially available kits (DiaSys Diagnostic Systems and Roche Diagnostics). Cholesteryl ester levels were calculated by subtracting free cholesterol from total cholesterol concentrations.

### Measurement of biliary lipid content

Biliary cholesterol concentrations were measured by GC as described above for fecal neutral sterol measurement following lipid extraction according to Bligh and Dyer (29). Biliary phospholipids were determined as described (30).

### Cholesterol flux measurements

To determine fractional cholesterol absorption, mice received an intravenous dose of 0.3 mg cholesterol-D_5_ (Medical Isotopes, Inc, Pelham, NH) in Intralipid (20%; Fresenius Kabi, Den Bosch, The Netherlands) and an oral dose of 0.6 mg cholesterol-D_7_ (Cambridge Isotope Laboratories, Inc.) in medium-chain triglyceride oil. Enrichment of cholesterol label in blood spots, taken from the tail at *t* = 0, 3, 6, 12, and 24 h, and daily thereafter for six additional days, was determined using GC-MS following trimethylsilylation of the cholesterol molecules. Fractional cholesterol absorption was calculated as described (31) and used to calculate transintestinal cholesterol excretion (TICE) according to the formula TICE = FNS − [(Chol_diet_ + Chol_bile_) × (1 − Fa)] (7), where FNS is fecal neutral sterols and Fa is fractional cholesterol absorption.

To determine cholesterol synthesis rates, mice received 2% [1-^13^C]acetate in the drinking water for 3 days. Blood spots were taken twice a day, starting 24 h after addition of [1-^13^C]acetate to the drinking water. Cholesterol synthesis rates were determined from the appearance of acetate-derived ^13^C atoms in cholesterol molecules retrieved from the blood spots as described (32).

### Statistics

Data in graphs are presented in Tukey box-and-whisker plots or line graphs with median and interquartile range, whereas data in tables are presented as median and range. Statistical analyses between two groups were performed by Mann-Whitney U nonparametric comparisons (GraphPad Software, San Diego, CA), whereas the Kruskal-Wallis H test followed by Conover post hoc analysis [BrightStat (33)] was used for multiple comparisons. Differences were considered statistically significant when *P* values were <0.05.

## RESULTS

### Inactivation of *Cyp2c70* by somatic gene editing induces a more human-like BA pool in mice

**Fig. 1.**
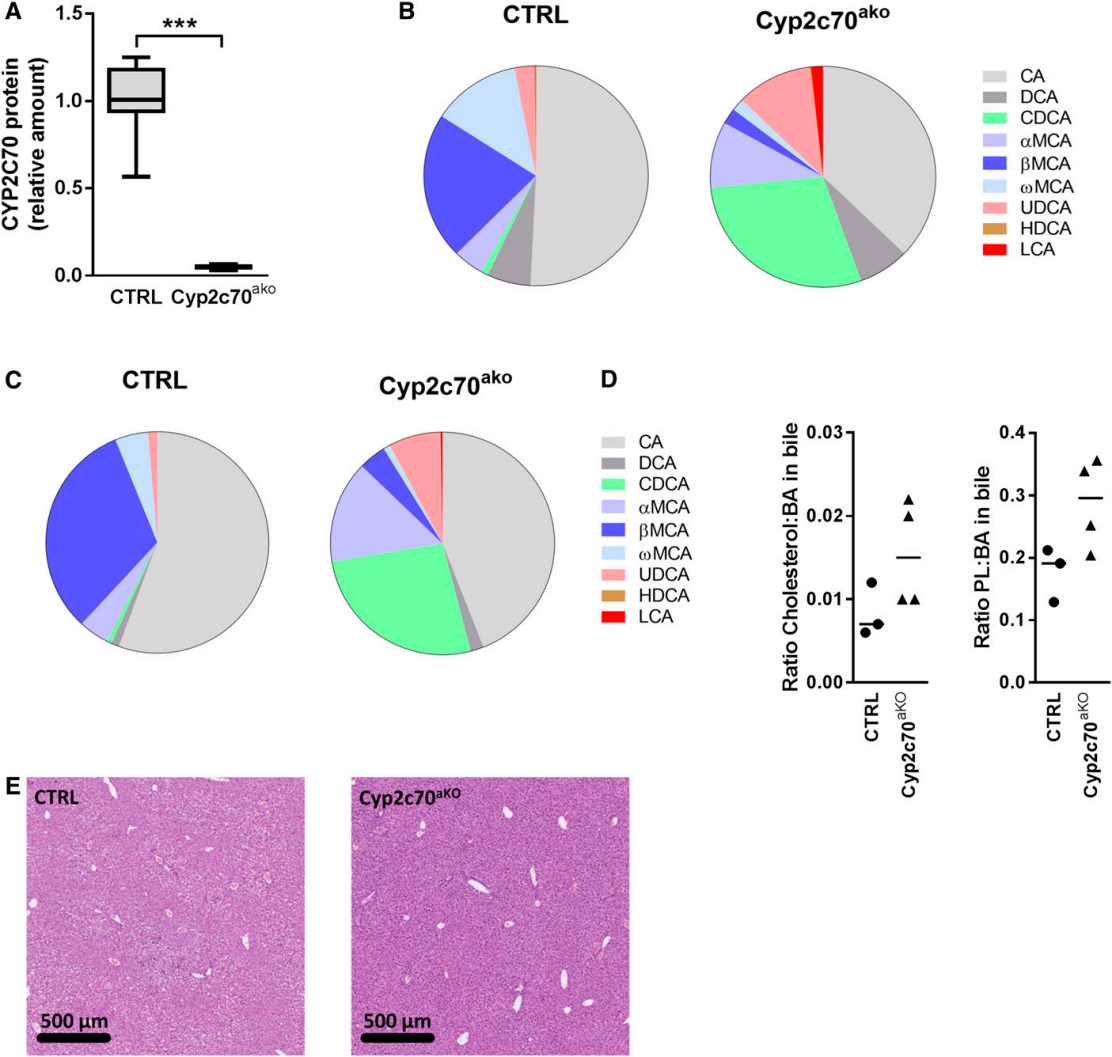
Acute knockout of *Cyp2c70* induces profound changes in BA composition. Hepatic protein levels of CYP2C70 were determined using targeted proteomics (A) (n = 6–8 animals per group). BA species distribution in plasma (B) (n = 8 animals per group) and bile (C) (n = 4 animals per group). Ratios of cholesterol:BAs and phospholipids:BAs in bile (D) (n = 3 animals per group). Liver sections stained with hematoxylin and eosin (E) (representative images of n = 8 animals per group). CTRL, Control. ***P < 0.001 (Mann-Whitney U test).

To acutely inactivate *Cyp2c70* in adult mouse livers, mice expressing Cas9 specifically in the liver were injected with adenovirus encoding a sgRNA targeting the first exon of *Cyp2c70*. After 4 weeks, the mice were euthanized and hepatic CYP2C70 protein levels were quantified using targeted proteomics (34). The amount of CYP2C70 was reduced by ∼95% in the L-Cas9tg mice injected with sgRNA-containing adenovirus [hereafter referred to as *Cyp2c70* acute knockout (*Cyp2c70*^ako^) mice] as compared with mice injected with a control virus, confirming highly efficient inactivation of the gene (**Fig. 1A**). A strong increase in the abundance of CDCA was observed in plasma (Fig. 1B; supplemental Tables S1, S2) and bile (Fig. 1C) of *Cyp2c70*^ako^ mice. Simultaneously, the amounts of βMCA and ωMCA were strongly decreased while, surprisingly, the amounts of αMCA were somewhat increased in *Cyp2c70*^ako^ mice. The fraction of UDCA in the BA pool also increased upon *Cyp2c70* inactivation, in line with observations in mice lacking the entire *Cyp2c* gene cluster described by Takahashi et al. (22). Furthermore, small amounts of LCA were present in the plasma of *Cyp2c70*^ako^ mice but not in controls. The contribution of CA was slightly reduced in *Cyp2c70*^ako^ mice. The conjugation of plasma (supplemental Tables S1, S2) and biliary BAs was not affected in *Cyp2c70*^ako^ mice. In bile, >99% of BAs were taurine-conjugated in both groups. In line with the superior capacity of CDCA to promote biliary lipid secretion compared with other BA species (35), the ratios of cholesterol:BAs and phospholipids:BAs were increased in bile of *Cyp2c70*^ako^ mice (Fig. 1D, supplemental Fig. S2).

Liver histology did not reveal major differences (Fig. 1E) between control and *Cyp2c70*^ako^ mice, although occasional immune cell infiltrations, likely representing a response toward the injected adenovirus particles, were observed in both groups. Concentrations of liver damage markers in plasma were somewhat higher in *Cyp2c70*^ako^ mice (**Table 2**), suggesting that the increased exposure to more cytotoxic CDCA and LCA may cause mild liver damage.

**TABLE 2.** Liver function markers in plasma

	Control (n = 4)	*Cyp2c70*^ako^ (n = 5)
Alanine aminotransferase (U/l)	62 (44–94)	278 (166–581)*^a^*
Aspartate aminotransferase (U/l)	175 (147–229)	1,012 (445–1582)*^a^*
Alkaline phosphatase (U/l)	117 (90–122)	224 (141–237)*^a^*
Albumin (g/l)	32 (31–34)	33 (32–34)
Gamma-glutamyl transferase (U/l)	<3	<3
Total bilirubin (μmol/l)	<3	<3

Values are presented as median (range) of male mice.

^a^ *P* < 0.05 versus control (Mann-Whitney U test).

**Fig. 2.**
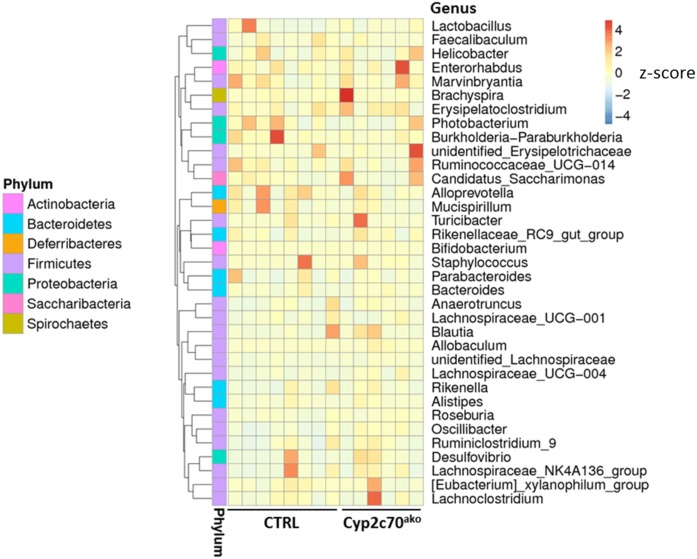
Impact of *Cyp2c70* ablation on microbiota. Microbial DNA was extracted from freshly frozen feces and 16S ribosomal DNA was sequenced. The abundance distribution of the 35 dominant genera among all samples is displayed in a species abundance heat map (n = 6–8 animals per group).

Because of the interactions between the intestinal microbiome and BAs, DNA was extracted from freshly frozen fecal pellets and microbial composition was analyzed using 16S ribosomal DNA sequencing. However, no major changes were observed in the composition of the microbiota between controls and *Cyp2c70*^ako^ mice (**Fig. 2**, supplemental Figure S3), indicating that the induced changes in BA composition did not translate into alterations of the colonic microbiota in our study.

### In vivo, CYP2C70 generates βMCA from CDCA in a two-step reaction

**Fig. 3.**
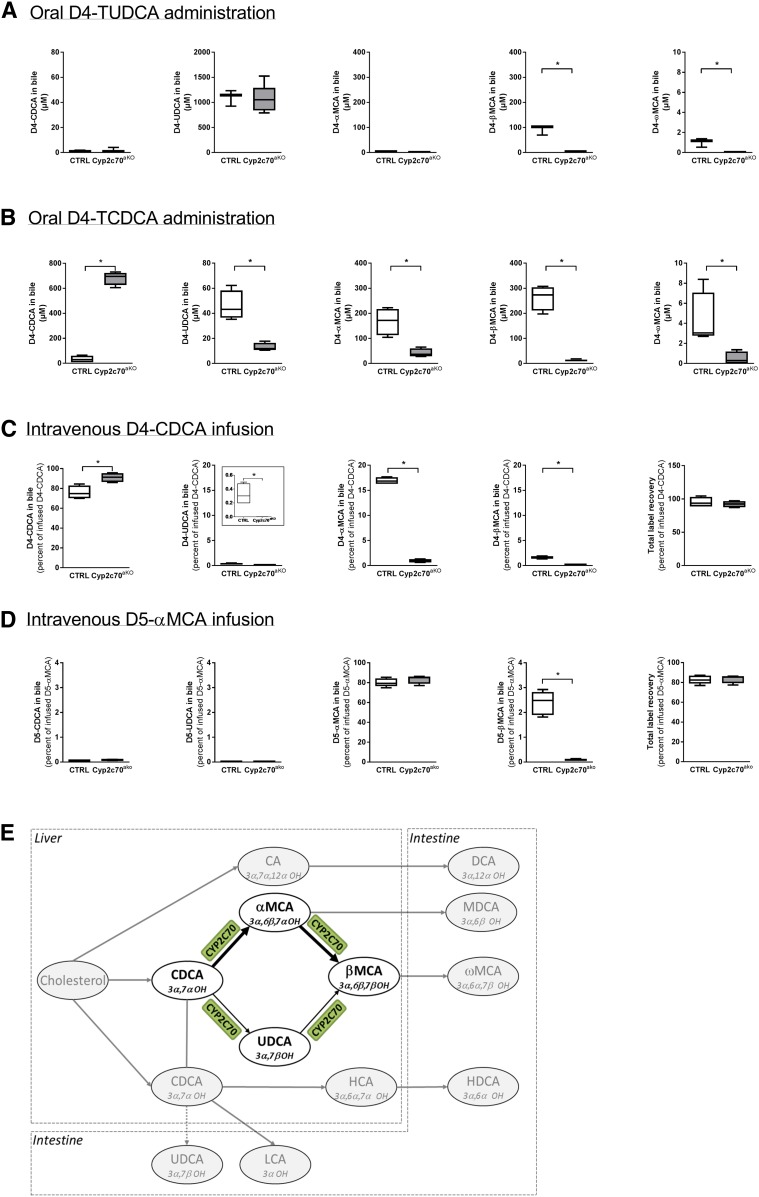
CYP2C70 is a 6β-hydroxylase as well as a C7-epimerase. Appearance of D4-labeled BAs in bile 16 h after oral administration of D4-TUDCA (A) or D4-TCDCA (B) as well as during intravenous infusion of D4-CDCA (C). Appearance of D5-labeled BAs in bile during intravenous infusion of D5-labeled αMCA. Schematic overview of the BA conversion reactions mediated by CYP2C70 (D) (n = 3–6 animals per group). *P < 0.05 (Mann-Whitney U test). TCDCA, taurochenodeoxycholic acid; MDCA, murodeoxycholic acid; HCA, hyocholic acid; HDCA, hyodeoxycholic acid; CTRL, control.

We performed a series of experiments to quantify the effects of *Cyp2c70* on the in vivo conversion of BAs. Because UDCA had been suggested as the substrate for the formation of βMCA in mice (22), we first assessed the in vivo conversion of this dihydroxylated BA into other species. D4-TUDCA (0.5 mg) was orally administered and bile was collected 16 h later. BAs cycle six to ten times per day in mice (36), which means that the orally administered D4-TUDCA had passed through the intestine and liver about five to eight times at the moment of bile collection and thus had repeatedly been exposed to the actions of hepatic and bacterial enzymes. Yet, the vast majority of administered UDCA remained unconverted (**Fig. 3A**). In support of 6β-hydroxylation activity of CYP2C70, some D4-βMCA was detected in the bile of control animals but not in the bile of *Cyp2c70*^ako^ mice. Notably, D4-βMCA concentrations were still an order of magnitude lower than those of D4-UDCA at 16 h after administration in control mice. In ad­dition, small amounts of labeled ωMCA could be detected in control animals only, while no D4-αMCA or D4-CDCA could be detected in any of the animals. The data from this experiment indicate that CYP2C70 does indeed catalyze the production of βMCA from UDCA. However, the low conversion rate makes it unlikely that this activity is sufficient to generate the large quantities of βMCA observed in mice. Therefore, we performed the same procedure following administration of D4-TCDCA (0.5 mg) in a separate group of mice. Nearly all D4-CDCA had been converted into other BA species 16 h after administration in control animals (Fig. 3B), showing that the in vivo turnover of CDCA is much faster than that of UCDA. High levels of D4-CDCA were, however, still detectable in the bile of *Cyp2c70*^ako^ mice, signifying the important role for this enzyme in the in vivo conversion of CDCA. In line with the C6 hydroxylation activity of CYP2C70, most of the converted tracer was detected in α- and βMCA in control animals, whereas concentrations of CDCA-derived D4-αMCA and D4-βMCA were strongly reduced in *Cyp2c70*^ako^ mice, i.e., ∼50 μM versus ∼450 μM in controls. Intriguingly, concentrations of D4-βMCA were markedly higher as compared with D4-αMCA in the bile of control animals, whereas the D4-βMCA to D4-αMCA ratio was reversed in the bile of *Cyp2c70*^ako^ mice. In addition, concentrations of CDCA-derived D4-UDCA were 4-fold lower in *Cyp2c70*^ako^ mice compared with controls, while low quantities of D4-ωMCA could be detected in the latter mice only.

To fully eliminate the impact of microbial enzymes on the administered tracers, we next assessed the conversion of BAs during a single passage through the liver. D4-CDCA was intravenously infused at a physiological rate while the bile produced by the liver was continuously collected. In *Cyp2c70*^ako^ mice, nearly all infused D4-CDCA was secreted into bile without being modified (Fig. 3C). In control mice, on the other hand, a substantial part, i.e., about 20%, was converted into other BA species by the liver. In line with the 6β-hydroxylase activity of CYP2C70, about 17% of infused D4-CDCA was converted into αMCA during a single passage through the liver of control animals, whereas only ∼1% of infused CDCA was secreted as αMCA in *Cyp2c70*^ako^ mice. Low, but detectable, amounts of CDCA-derived D4-UDCA were found in the bile of control but not *Cyp2c70*^ako^ mice. We also found D4-labeled βMCA in the bile of control mice, while no D4-βMCA was present in the bile of *Cyp2c70*^ako^ mice. In line with the consensus that ωMCA is a secondary BA (37), no D4-ωMCA was detected in any of the mice in this experiment. As expected, no D4-label was detected in the C12-hydroxylated BAs, CA and DCA (data not shown). Furthermore, cumulative recovery of infused D4-label in biliary BAs was nearly 100% (Fig. 3C).

The data from the experiments above indicate that CYP2C70 possesses epimerase activity that may contribute to the formation of βMCA from αMCA. Therefore, we performed an additional set of infusion experiments to study the conversion of D5-αMCA. Indeed, about 2–3% of intravenously infused D5-αMCA was selectively converted into βMCA, but not into other BA species, during one passage through the liver in control animals, whereas such conversion did not take place in *Cyp2c70*^ako^ mice (Fig. 3D). Because the BA pool efficiently cycles between liver and intestine, this conversion rate will generate substantial quantities of βMCA in the murine BA pool.

Taken together, our data demonstrate that CYP2C70 exerts epimerase activity toward the C7 hydroxyl group of αMCA and CDCA. This property of CYP2C70 leads to the conversion of αMCA into βMCA and, to a lesser extent, CDCA into UDCA (Fig. 3E). Although CYP2C70 indeed also catalyzes the production of βMCA from UDCA by its 6β-hydroxylase activity (22), our data indicate that the two-step conversion of CDCA into βMCA, generating αMCA as an intermediate metabolite, is quantitatively more important in vivo.

### Impact of inactivation of *Cyp2c70* on the response to pharmacological FXR activation

**Fig. 4.**
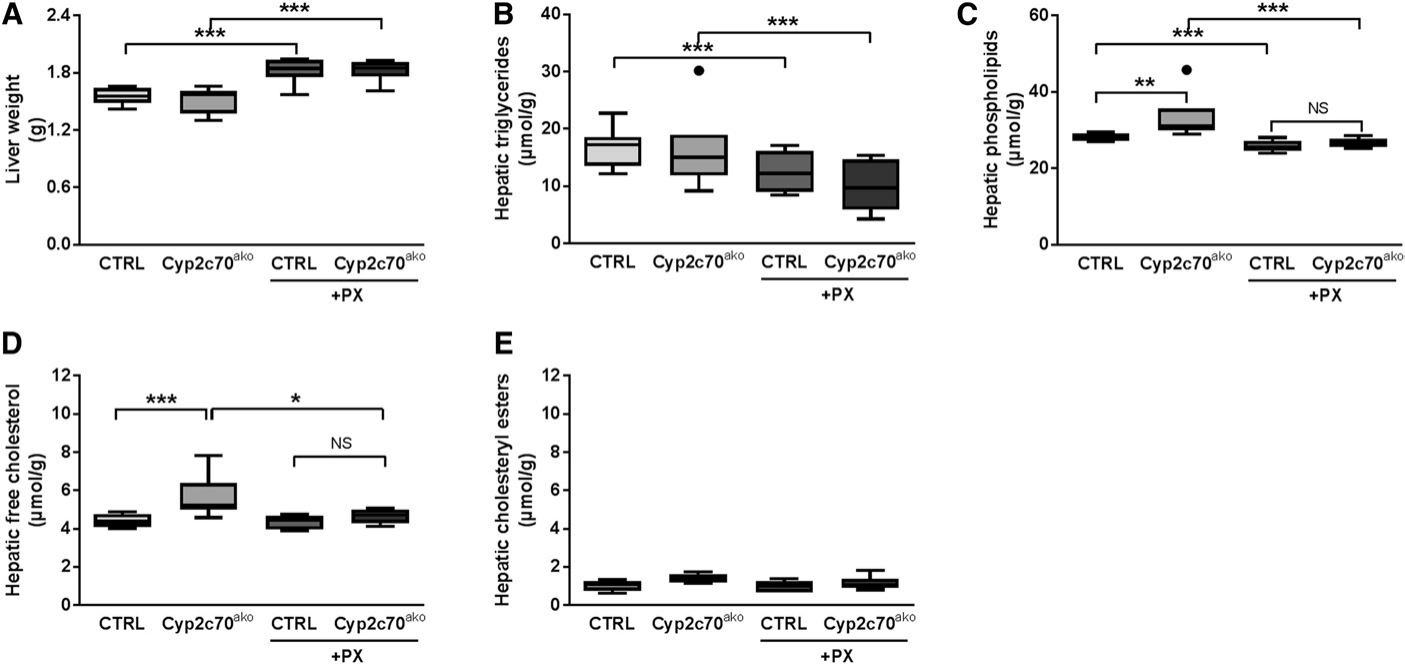
Increased hepatic cholesterol content in *Cyp2c70*^ako^ mice is reversed by pharmacological FXR activation. Liver weight (A), hepatic content of triglycerides (B), phospholipids (C), free cholesterol (D), and cholesteryl esters (E) in chow-fed *Cyp2c70*^ako^ mice and controls (CTRL) as well as after treatment with the FXR agonist PX (10 mg/kg/day) in the diet for 18 days (n = 6–8 animals per group). *P < 0.05, **P < 0.01, ***P < 0.001 (Kruskal-Wallis H test, followed by Conover post hoc comparisons). CTRL, control.

We have previously shown that pharmacological activation of FXR in C57BL/6J mice with PX20606 (PX) results in a BA pool that is dominated by βMCA and strongly enhanced cholesterol removal from the body via stimulation of TICE (7). The shift in composition of the murine BA pool toward MCAs upon FXR activation evidently hampers translation of observed metabolic responses to the human situation. As a proof of principle, we therefore evaluated the effects of FXR activation in *Cyp2c70*^ako^ mice on parameters of cholesterol metabolism by feeding them a chow-based diet with or without PX (10 mg/kg/day) for 18 days. No significant differences in plasma lipid levels were observed between the groups (**Table 3**). Liver weights were not altered by *Cyp2c70* inactivation, but were slightly increased in mice receiving the FXR agonist (**Fig. 4A**). Likewise, hepatic triglyceride contents were not affected in *Cyp2c70*^ako^ mice but, as expected, were reduced in the groups treated with PX (Fig. 4B). Hepatic phospholipid and cholesterol contents were slightly increased upon *Cyp2c70* inactivation in chow-fed mice, but this effect was reversed upon PX treatment (Fig. 4C–E).

**TABLE 3.** Hepatic lipid content and liver function parameters measured in plasma

	Vehicle		PX	
	Control (n = 8)	*Cyp2c70*^ako^ (n = 6)	Control (n = 6)	*Cyp2c70*^ako^ (n = 7)
Total cholesterol (mmol/l)	2.71 (2.37–3.63)	2.75 (2.57–2.84)	2.49 (2.12–2.80)	2.40 (1.61–3.09)
Free cholesterol (mmol/l)	0.98 (0.88–1.43)	1.19 (0.96–1.52)	1.00 (0.79–1.31)	0.90 (0.66–1.42)
Cholesteryl esters (mmol/l)	1.72 (1.33–2.26)	1.58 (1.05–1.75)	1.33 (1.21–1.75)	1.41 (0.95–1.68)
Triglycerides (mmol/l)	1.27 (0.66–2.89)	1.61 (0.71–2.10)	0.95 (0.72–1.77)	1.12 (0.61–1.54)
Free fatty acids (mmol/l)	0.20 (0.08–0.36)	0.17 (0.15–0.74)	0.32 (0.15–0.56)	0.28 (0.22–0.52)
Alanine aminotransferase (U/l)	76 (42–141)	149 (91–180)*^a^*	77 (21–143)	145 (56–293)
Aspartate aminotransferase (U/l)	32 (18–64)	49 (36–60)*^a^*	24 (20–38)	49 (17–86)*^a^*

Values are presented as median (range) of male mice.

^a^ *P* < 0.05 versus control (Kruskal-Wallis H test, followed by Conover post hoc comparisons).

**Fig. 5.**
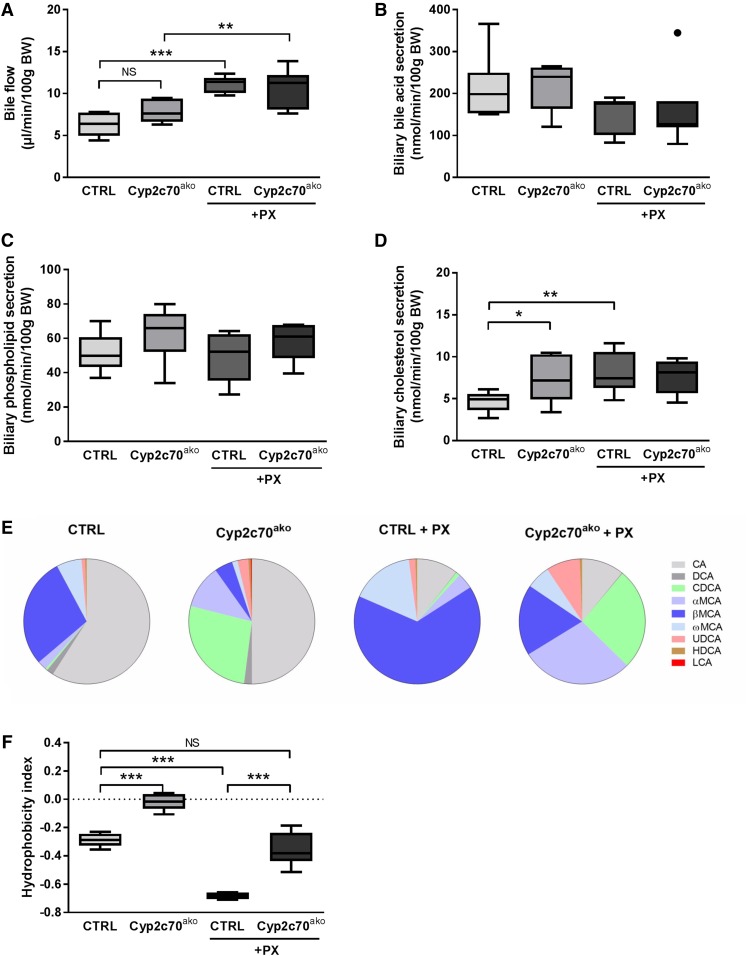
Hydrophobic BA pool in *Cyp2c70*^ako^ stimulates biliary cholesterol secretion. Gallbladders of *Cyp2c70*^ako^ mice and controls with or without PX (10 mg/kg/day) were cannulated and bile flow (A), biliary secretion rates of total BAs (B), phospholipids (C), and cholesterol (D) were determined. The composition of biliary BAs (E) was determined using LCMS and used to calculate the hydrophobicity index according to Heuman (19) (see text) (F) (n = 6–8 animals per group). *P < 0.05, **P < 0.01, ***P < 0.001 (Kruskal-Wallis H test followed by Conover post hoc comparisons). CTRL, control.

**Fig. 6.**
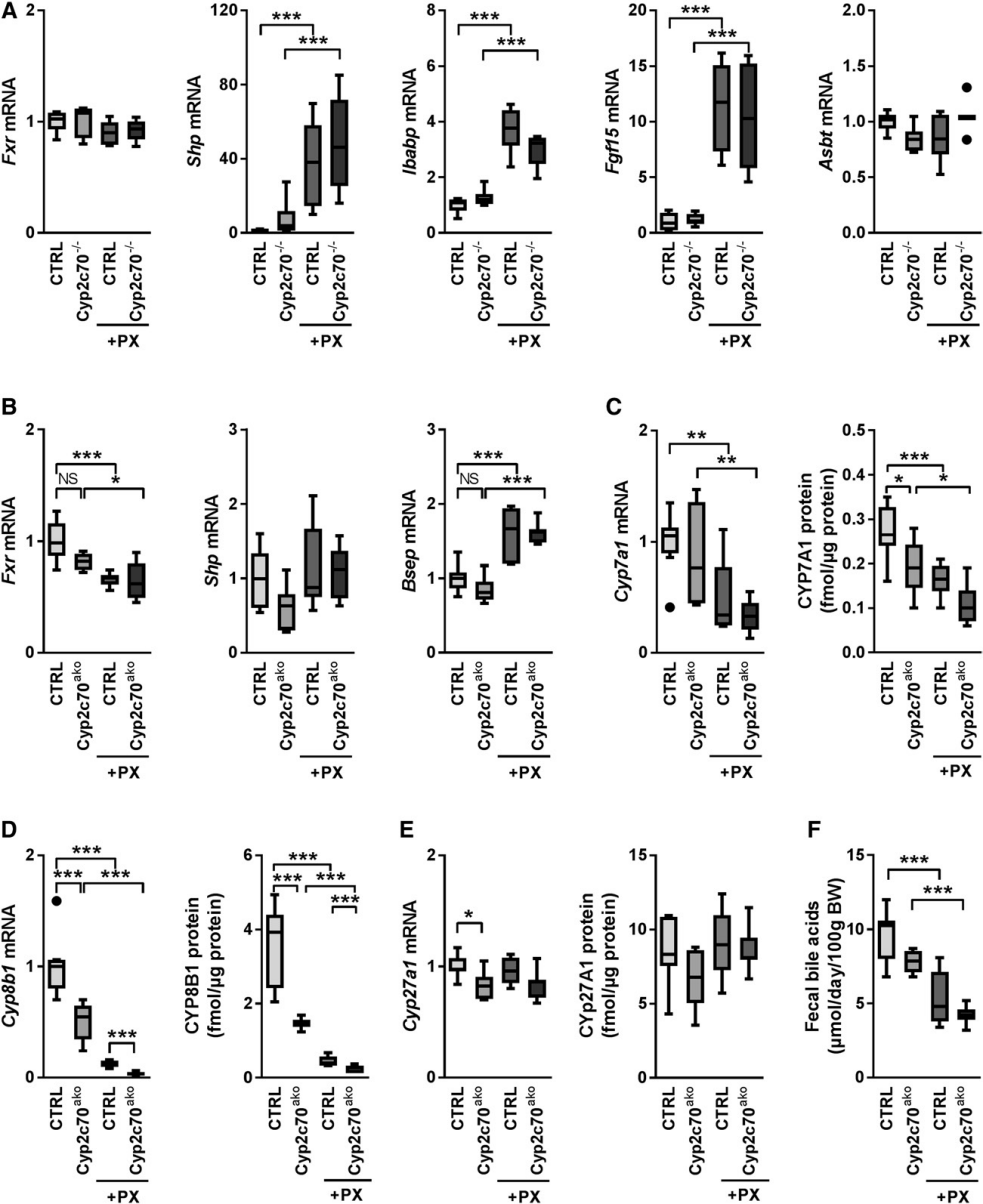
BA signaling and synthesis in *Cyp2c70*^ako^ mice. Ileal (A) and hepatic (B) mRNA expression levels of genes involved in BA signaling were determined by real-time qPCR on reverse transcribed RNA isolated from freshly frozen tissue obtained from *Cyp2c70*^ako^ mice and controls with or without PX (10 mg/kg/day). Hepatic mRNA and protein expression of CYP7A1 (C), CYP8B1 (D), and CYP27A1 (E) were determined by qPCR and targeted proteomics, respectively. Fecal BA excretion (F), which equals hepatic BA synthesis in steady-state conditions, was determined by gas-LC. All data are normalized to the housekeeping gene, cyclophilin, and further normalized to the mean of the control group, i.e., chow-fed control mice (n = 6–8 animals per group). *P < 0.05, **P < 0.01, ***P < 0.001 (Kruskal-Wallis H test followed by Conover post hoc comparisons). CTRL, control.

Gallbladder cannulation was performed to assess the effects of FXR activation in the setting of *Cyp2c70* inactivation on bile formation, hepatic BA secretion, and biliary lipid content. Bile flow was not significantly altered upon *Cyp2c70* inactivation, but was ∼1.7-fold and ∼1.4-fold higher upon PX treatment in control and *Cyp2c70*^ako^ mice, respectively (**Fig. 5A**). Biliary BA and phospholipid secretion rates were, however, not significantly altered in any of the groups (Fig. 5B, C). In PX-treated animals, this was due to reduced concentrations of these components in the bile (supplemental Fig. S4A, B). In control mice, FXR activation resulted in increased biliary cholesterol secretion (Fig. 5D). Secretion of cholesterol into the bile was ∼1.5-fold higher in chow-fed *Cyp2c70*^ako^ mice as compared with controls, but was not further enhanced by FXR stimulation (Fig. 5D). The strongly elevated concentrations of CDCA in the bile of *Cyp2c70*^ako^ mice (supplemental Table S3), mainly at the expense of βMCA, translated into a markedly higher hydrophobicity index of the biliary BAs as calculated according to Heuman (19) (Fig. 5E, F). As expected (7), FXR stimulation induced a strong increase in the percentage of MCAs at the expense of CA and DCA in the bile of control mice. Administration of PX to *Cyp2c70*^ako^ mice also induced a decrease of CA and DCA in bile, whereas the relative abundance of CDCA remained more or less unaffected. Instead, a surprising increase in MCAs was observed in PX-treated versus vehicle-treated *Cyp2c70*^ako^ mice (Fig. 5E). Conceivably, this may be explained by a reduction of BA pool size and a relatively more efficient enterohepatic recycling of hydrophilic BAs upon FXR activation. In support of this hypothesis, the percentage of biliary secreted MCAs that was lost with feces decreased upon PX treatment (supplemental Fig. S4C). Furthermore, no increase in hepatic CYP2C70 protein levels was detected in PX-treated versus chow-fed *Cyp2c70*^ako^ mice (data not shown), excluding the possibility of an upregulation of residual *Cyp2c70* expression upon FXR activation. Nevertheless, BAs present in the bile of PX-treated *Cyp2c70*^ako^ mice were considerably less hydrophilic as compared with those in the bile of PX-treated controls (Fig. 5F). The increased amounts of the potent FXR agonist CDCA in the bile of *Cyp2c70*^ako^ mice did not, however, translate into significant inductions of the FXR target genes *Nr0b2* (*Shp*), fatty acid binding protein 6 (*Fabp6*, *Ibabp*), or *Fgf15* in the terminal ileum, whereas PX induced the expression of these genes with equal strength in both groups (**Fig. 6A**). Ileal expression of *Slc10a2* (*Asbt*) was similar in all the groups (Fig. 6A). Also, in the liver, no differences in mRNA expression of *Nr1h4* (*Fxr)* and its target genes, *Shp* and *Abcb11* (*Bsep*), were observed between chow-fed *Cyp2c70*^ako^ mice and controls, whereas treatment with the FXR agonist had similar effects on mRNA expression levels of these genes in both groups (Fig. 6B). Genes encoding transporters involved in hepatic BA uptake, *Slc10a1* (*Ntcp*) and *Slco1a1* (*Oatp1a1*), were downregulated in *Cyp2c70*^ako^ mice as well as by pharmacological FXR stimulation, but were not further reduced in *Cyp2c70*^ako^ mice treated with the FXR agonist (supplemental Fig. S5). In chow-fed *Cyp2c70*^ako^ mice, hepatic mRNA expression of *Cyp7a1*, encoding cholesterol-7α hydroxylase, the first and rate-controlling enzyme in the classical BA synthesis pathway, showed considerable variation and was not significantly different from WT littermates (Fig. 6C, left panel). Protein levels of CYP7A1 in the liver were, however, significantly decreased (Fig. 6C, right panel). Hepatic mRNA expression of sterol-12α hydroxylase (*Cyp8b1*), essential for CA synthesis, was ∼50% decreased (Fig. 6D, left panel), while CYP8B1 protein expression was ∼60% lower in *Cyp2c70*^ako^ mice compared with controls (Fig. 6D, right panel). Expression of both of these enzymes was reduced in groups treated with the FXR agonist. Hepatic mRNA expression of *Cyp27a1*, encoding the enzyme mediating the first step in the alternative BA synthesis pathway that produces CDCA, was reduced in chow-fed *Cyp2c70*^ako^ mice (Fig. 6E, left panel), but was not affected by PX. CYP27A1 protein levels only displayed an insignificant tendency toward a decrease in chow-fed *Cyp2c70*^ako^ mice (Fig. 6E, right panel). Expression of the gene encoding 25-hydroxycholesterol 7α-hydroxylase (*Cyp7b1*), another important enzyme in the alternative BA synthesis pathway, remained unchanged in *Cyp2c70*^ako^ mice, but was reduced ∼50% in the groups receiving PX (data not shown). In line with reduced amounts of CYP7A1 protein, fecal BA excretion, reflecting hepatic BA synthesis, tended to be lower in chow-fed *Cyp2c70*^ako^ mice, but this difference did not reach statistical significance (Fig. 6F). Treatment with PX reduced fecal BA excretion in *Cyp2c70*^ako^ and control mice, in keeping with repression of BA synthesis upon pharmacological FXR stimulation (38).

**Fig. 7.**
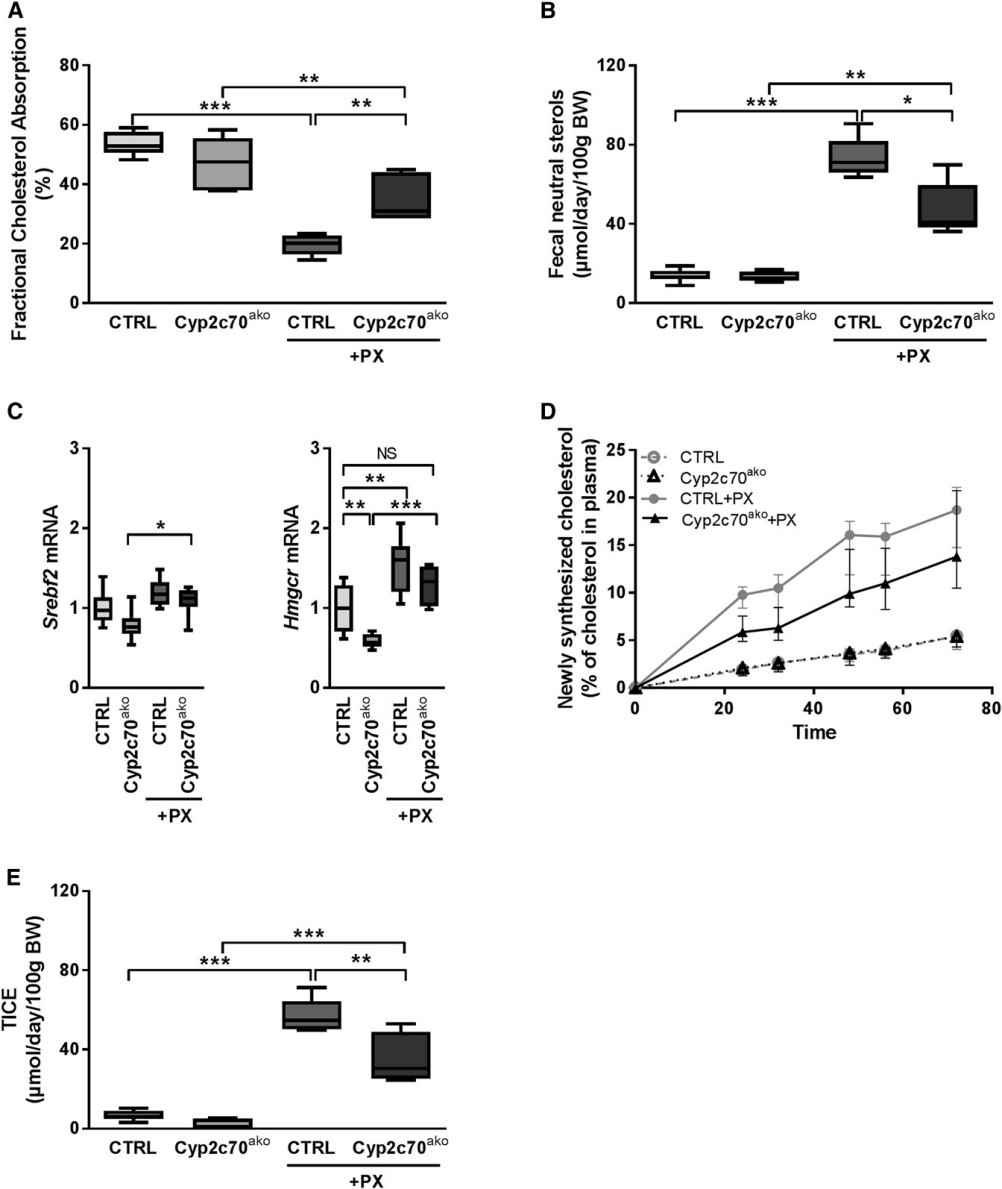
The altered BA pool composition in *Cyp2c70*^ako^ mice impacts on FXR activation-induced changes in cholesterol homeostasis. A: Fractional cholesterol absorption in *Cyp2c70*^ako^ mice and controls with or without PX (10 mg/kg/day) determined by appearance of orally administered cholesterol-D_7_ and intravenously administered cholesterol-D_5_ in the blood. B: Fecal neutral sterol excretion. C: Hepatic mRNA expression of genes involved in cholesterol synthesis. D: Cholesterol synthesis determined by incorporation of [1-^13^C]acetate. E: TICE calculated as described in the Methods section (n = 6–8 animals per group). *P < 0.05, **P < 0.01, ***P < 0.001 (Kruskal-Wallis H test followed by Conover post hoc comparisons). CTRL, control.

In accordance with data reported previously (7), PX treatment markedly reduced fractional cholesterol absorption in control mice (**Fig. 7A**), most conceivably due to the inferior cholesterol-solubilizing properties of the MCA-dominated hydrophilic BA pool. The more hydrophobic BA pool in chow-fed *Cyp2c70*^ako^ mice did not result in higher fractional cholesterol absorption under control conditions, but did enhance cholesterol absorption upon FXR stimulation. Fecal excretion of neutral sterols, i.e., cholesterol and its bacterial metabolites, was not altered in *Cyp2c70*^ako^ mice under control conditions (Fig. 7B). However, the prominent stimulation of fecal neutral sterol secretion by PX was significantly blunted in *Cyp2c70*^ako^ mice compared with controls. The augmented fecal cholesterol loss in PX-treated control mice was accompanied by a compensatory increase in cholesterol synthesis, as indicated by increased hepatic mRNA expression of HMG-CoA reductase (*Hmgcr*) as well as by direct quantification of cholesterol synthesis using incorporation of the precursor [1-^13^C]acetate into cholesterol molecules (Fig. 7C, D) (39). Lower mRNA expression of *Hmgcr* in livers of chow-fed *Cyp2c70*^ako^ mice compared with controls did not translate into reduced cholesterol synthesis as determined by the incorporation of labeled acetate. The induction of cholesterol synthesis upon FXR activation in *Cyp2c70*^ako^ mice tended to be lower as compared with controls, but the difference did not reach statistical significance due to large inter-individual variations within the groups (Fig. 7D).

In mice, cholesterol removal via the TICE pathway (40–42) is strongly stimulated by pharmacological FXR activation, which is conceivably attributable to stimulatory actions of MCAs on the intestinal sterol efflux transporter ABCG5/G8 (7). The quantified cholesterol fluxes were therefore used to calculate the origin of fecal neutral sterols. In the absence of pharmacological FXR stimulation, TICE amounted to about 7 μmol/day/100 g body weight in control animals (Fig. 7E). Yet, TICE appeared less active in *Cyp2c70*^ako^ mice under chow-fed conditions. As expected, PX treatment strongly induced TICE in control mice. The PX-induced stimulation of TICE was less pronounced in *Cyp2c70*^ako^ mice, likely due to the strong reduction of rodent-specific MCAs.

## DISCUSSION

In this study, we have generated a mouse model with a more human-like BA pool composition by inactivating *Cyp2c70* in the adult mouse liver using somatic genome editing. We employed this model to delineate the actions of CYP2C70 on BAs in vivo. Stable isotope tracer studies demonstrated that CYP2C70 mainly produces βMCA from CDCA by catalyzing a two-step reaction. Parameters of cholesterol homeostasis were differentially affected in *Cyp2c70*^ako^ mice compared with controls upon pharmacological FXR activation, underscoring the impact species-specific BA metabolism on the outcome of pharmacological interventions that target BA signaling or impact BA production.

The changes in BA composition did not induce major alterations in microbiome composition. This is surprising considering the known interactions between BAs and intestinal bacteria (43), but may be related to the fact that the change in BA composition was only induced during adulthood, i.e., when intestinal bacterial populations have been firmly established, or to the relatively short duration of the study. In addition, fecal microbiota composition may not accurately reflect the microbiota present in the distal small intestine and cecum (44), where bacteria are exposed to higher concentrations of BAs and are therefore more likely to be affected by the altered BA composition in *Cyp2c70*^ako^ mice.

In vitro experiments using the human hepatocyte cell line HepG2 transfected with murine *Cyp2c70* had previously indicated that this enzyme mediates the production of αMCA from CDCA and βMCA from UDCA (22). Our novel mouse model, in combination with stable isotope labeling procedures, now allowed us to accurately quantify the enzymatic activities of CYP2C70 in vivo. Data from these experiments demonstrate that CYP2C70 exerts a dual function, 6β-hydroxylation as well as epimerization of the C7 hydroxyl group from the α to the β configuration. CYP2C70 thereby not only mediates the conversion of CDCA into αMCA and UDCA into βMCA, but also mediates the conversion of αMCA into βMCA and CDCA into UDCA. The conversion rate of CDCA into αMCA is the highest, followed by αMCA into βMCA and UDCA into βMCA, while only miniscule amounts of CDCA are converted into UDCA by CYP2C70. The substantially different conversion rates of the reactions mediated by CYP2C70 may explain why Takahashi et al. (22) did not detect CDCA-derived βMCA in their in vitro experiments with the *Cyp2c70*-transfected HepG2 cells when D4-CDCA was added to the culture medium. Most likely, the high affinity of CYP2C70 for CDCA, combined with the considerable amounts of this BA present in the culture medium, prevented the second reaction, conversion of CDCA-derived αMCA into βMCA, from happening. The low but clearly detectable amounts of D4-UDCA present in bile after intravenous infusion of D4-CDCA in control animals demonstrate that UDCA is a primary BA in mice. This interpretation is supported by a study performed by Sayin et al. (21), who found UDCA in germ-free mice.

An issue that remains unanswered concerns the origin of the (relatively small) amounts of MCAs in the BA pool of *Cyp2c70*^ako^ mice in this study. It is conceivable that some functional CYP2C70 was still present in the liver due to hepatocytes that escaped from genome editing or as a consequence of incomplete inactivation of the *Cyp2c70* gene resulting from in-frame mutations. Although CYP2C70 protein was virtually undetectable in *Cyp2c70*^ako^ mice, incomplete ablation of protein expression is not uncommon when using CRISPR/Cas9-mediated somatic genome editing in the livers of adult mice (23). Extrahepatic expression of CYP2C70 could also contribute to MCA production. We detected *Cyp2c70* mRNA in the ileum (Ct-values ∼33), but levels were ∼1,000-fold lower than in the liver. The likelihood that significant amounts of preexisting MCAs were still present 4 weeks after injection with the virus encoding the sgRNA directed against *Cyp2c70* seems fairly low given the fractional turnover rate of CA in mice of ∼0.3–0.5 pools/day (36). However, the possibility cannot be fully excluded because the turnover of MCAs may be slower than that of CA. Several attempts have been made to identify the enzyme(s) involved in MCA production. Mice lacking seven genes of the *Cyp3a* cluster, including *Cyp3a11*, produce normal amounts of MCAs (45), whereas mice lacking the entire *Cyp2c* gene cluster no longer produce these BAs (22). The fact that human hepatocytes become capable of producing MCAs when transfected with *Cyp2c70* does not, however, exclude the possibility that other members of the Cyp2c family possess overlapping activities with CYP2C70. Full-body *Cyp2c70*-deficient mice are required to demonstrate whether or not CYP2C70 is the only enzyme capable of producing MCAs in mice.

To explore the physiological consequences of the humanized BA pool, we quantified cholesterol fluxes in *Cyp2c70*^ako^ mice and controls upon pharmacological FXR stimulation. Intriguingly, the bile formation process, as such, appeared to be unaffected by *Cyp2c70* deletion: bile flow and its major driver, i.e., biliary BA secretion, were highly similar in control and *Cyp2c70*^ako^ mice. Yet, the more hydrophobic BA pool in *Cyp2c70^ako^* mice did stimulate cholesterol mobilization at the hepatocytic canalicular membranes, resulting in ∼50% increased biliary cholesterol secretion. The higher abundance of CDCA in the circulating BA pool of *Cyp2c70*^ako^ mice did not appear to result in stronger activation of hepatic FXR, as expression of its target genes in the liver was not increased. Because BA pool size was not determined in this study, it cannot be fully excluded that this can be explained by a reduction of the size of the BA pool in *Cyp2c70*^ako^ mice. Biliary BA secretion rates were, however, very similar between WT and *Cyp2c70*^ako^ mice and fecal BA excretion also did not differ significantly between the groups, which makes it unlikely that BA pool size is substantially affected by inactivation of *Cyp2c70*. Reduced hepatic uptake of BAs due to lower expression of *Ntcp* and *Oatp1a1* may underlie the somewhat surprising absence of additional FXR activation in the livers of *Cyp2c70*^ako^ mice compared with controls as well as the mildly increased plasma BA levels in these animals (supplemental Table S2). Expression of *Ntcp* and *Oatp1a1* is known to be inhibited by SHP. However, compared with controls, *Shp* expression remained unchanged in *Cyp2c70*^ako^ mice. The reason for the reduced expression of *Ntcp* and *Oatp1a1* therefore remains to be elucidated. Hepatic CYP7A1 protein levels were significantly reduced at the moment of euthanization, but fecal BA excretion only showed a tendency toward a moderate reduction. This indicates that BA synthesis, i.e., average CYP7A1 activity over the entire day, was not majorly affected upon *Cyp2c70* inactivation. Hepatic CYP8B1 protein as well as mRNA were reduced in *Cyp2c70*^ako^ mice to a similar extent, suggesting that regulation took place mainly at the transcriptional level. Our data suggest that the reduced gene expression of *Cyp8b1* is due to mechanisms unrelated to activation of hepatic FXR or FGF15 from the intestine. Further studies are required to clarify the mechanisms underlying the apparent gene-specific regulation of the genes involved in BA synthesis and transport mentioned above.

The relatively hydrophobic BA pool composition in *Cyp2c70^ako^* mice did not translate into augmented intestinal fractional cholesterol absorption. The mice were, however, fed a standard low-cholesterol diet. It might well be that increasing dietary cholesterol content uncovers an enhanced cholesterol absorption capacity in *Cyp2c70*^ako^ mice. Under conditions of FXR stimulation, *Cyp2c70*^ako^ mice did show higher fractional cholesterol absorption than controls. In line with previously reported data (7), FXR stimulation resulted in a strong increase of fecal cholesterol disposal in control mice, mainly due to increased net transport of cholesterol by the intestinal cells into the lumen, i.e., TICE. In *Cyp2c70^ako^* mice, the stimulation of fecal cholesterol disposal and TICE by the FXR agonist were clearly blunted. Moreover, TICE already tended to be lower in *Cyp2c70*^ako^ mice compared with controls in the absence of pharmacological FXR activation. These data are in agreement with our previous observations that hydrophilic BAs not only reduce cholesterol absorption efficiency, but also increase active secretion of cholesterol by enterocytes into the intestinal lumen (7).

In addition to the presence of rodent-specific BA species in mice, conjugation of BAs also differs between mice and humans. In normal mice as well as in our *Cyp2c70*^ako^ mice, BAs are almost exclusively conjugated to taurine, whereas taurine- as well as glycine-conjugated BAs are found in humans. However, the physiological consequences of this difference are most conceivably limited. The hydrophobicity of glycine-conjugated BAs is only modestly higher compared with that of those conjugated to taurine (19) and the impact on FXR activation is minimal (46).

In conclusion, we have generated a mouse model with a more human-like BA metabolism by targeting *Cyp2c70* in adult mice that is anticipated to improve translation of preclinical data to the human situation. In vivo studies with stable isotopically labeled BA tracers revealed that, in vivo, βMCA is mainly produced from CDCA by two CYP2C70-catalyzed reactions. The first reaction generates αMCA by the addition of a hydroxyl group to the C6 of CDCA. The second CYP2C70-mediated reaction is epimerization of the C7 hydroxyl group of αMCA from the α to the β configuration, yielding βMCA. Furthermore, the presence of a human-like BA pool composition in *Cyp2c70*^ako^ mice resulted in blunted FXR-induced cholesterol disposal due to decreased TICE, which emphasizes the importance to carefully consider the consequences of species-specific (BA) metabolism in preclinical studies.

## Supplementary Material

Supplemental Data
