# Supplementary material for: A human-like bile acid pool induced by deletion of hepatic Cyp2c70 modulates effects of FXR activation in mice
Source: J Lipid Res. 2019 Sep 10;61(3):291–305. doi: 10.1194/jlr.RA119000243 (PMC7053831; doi:10.1194/jlr.RA119000243)
Supplement: Supplemental Data [file supp_61_3_291__index.html]

A Human-like Bile Acid Pool Induced by Deletion of Cyp2c70 Modulates Effects of Farnesoid X Receptor Activation in Mice — Effects of acute inactivation of hepatic Cyp2c70 in mice — A human-like bile acid pool induced by deletion of hepatic Cyp2c70 modulates effects of FXR activation in mice — Supplemental Data 

# A human-like bile acid pool induced by deletion of hepatic *Cyp2c70* modulates effects of FXR activation in mice

## Supplemental Data

- Supplemental data - Supplemental data to be published online
