# Supplementary material for: A human-like bile acid pool induced by deletion of hepatic Cyp2c70 modulates effects of FXR activation in mice
Source: J Lipid Res. 2019 Sep 10;61(3):291–305. doi: 10.1194/jlr.RA119000243 (PMC7053831; doi:10.1194/jlr.RA119000243)
Supplement: Supplemental Data [file supp_RA119000243_154098_2_supp_389839_pxckjw.pdf]

Supplemental data

A Human-like Bile Acid Pool Induced by Deletion of Hepatic Cyp2c70 Modulates Effects of Farnesoid X Receptor Activation in Mice

Jan Freark de Boer[1,2,*], Esther Verkade[2,*], Niels L. Mulder[2], Hilde D. de Vries[1], Nicolette Huijkman[2,3], Martijn Koehorst[1], Theo Boer[1], Justina C. Wolters[2], Vincent W. Bloks[2], Bart van de Sluis[2,3], Folkert Kuipers[1,2]

*****Equal contribution

Affiliations:
Departments of [1]Laboratory Medicine and [2]Pediatrics, University of Groningen, University Medical Center Groningen, Groningen, The Netherlands
[3]iPSC/CRISPR Center Groningen, University of Groningen, University Medical Center Groningen, Groningen, The Netherlands

Supplemental Table S1. Relative abundance of individual bile acid species in plasma

Supplemental Table S2. Concentrations of individual bile acid species in plasma

Supplemental Table S3. Concentrations of individual bile acid species in bile

Supplemental Figure S1. Expression cassette of single-guide RNA targeting *Cyp2c70*

Supplemental Figure S2. Bile composition

Supplemental Figure S3. Impact of *Cyp2c70* ablation on microbiota

Supplemental Figure S4. Concentrations of bile acids and phospholipids in bile and fecal loss of muricholic acids

Supplemental Figure S5. Hepatic mRNA expression of bile acid uptake transporters

Supplemental Table S1. Relative abundance of individual bile acid species in plasma

Bile acid	CTRL (n=8)	*Cyp2c70*^{ako} (n=8)	p-value
LCA (%)	0.0 [0.0-0.4]	1.2 [0.6-2.3]	0.0007
DCA (%)	3.7 [1.4-5.5]	4.1 [1.5-6.8]	0.645
CDCA (%)	0.3 [0.0-1.4]	8.6 [3.4-17.3]	0.0009
UDCA (%)	1.1 [0.3-2.8]	3.6 [0.7-4.1]	0.028
HDCA (%)	0.0 [0.0-0.4]	0.0 [0.0-0.3]	0.225
CA (%)	7.0 [1.8-18.2]	6.3 [2.7-10.5]	0.879
αMCA (%)	0.0 [0.0-0.8]	0.6 [0.2-1.0]	0.022
βMCA (%)	5.9 [0.9-16.5]	0.6 [0.1-1.1]	0.0006
ωMCA[a] (%)	5.1 [1.0-15.9]	0.7 [0.2-2.5]	0.001
T-LCA (%)	0.0 [0.0-0.0]	0.4 [0.3-0.7]	0.0006
T-DCA (%)	2.5 [0.0-4.9]	3.1 [1.3-5.3]	0.574
T-CDCA (%)	0.7 [0.0-1.2]	19.0 [13.6-29.1]	0.0009
T-UDCA (%)	1.5 [0.5-2.5]	4.8 [2.6-8.0]	0.0002
T-HDCA (%)	0.2 [0.0-0.8]	0.3 [0.0-0.4]	0.848
T-CA (%)	43.8 [21.4-64.1]	32.3 [17.6-39.4]	0.021
T-αMCA (%)	4.2 [2.3-5.6]	8.6 [5.0-13.8]	0.0006
T-βMCA (%)	16.1 [9.4-22.1]	1.5 [1.0-3.1]	0.0002
T-ωMCA (%)	6.2 [3.5-10.0]	0.8 [0.4-1.8]	0.0002
Conjugated bile acids (%)	74.8 [43.7-93.7]	71.6 [55.8-89.7]	0.721

Relative abundance of bile acid species in plasma of male mice presented as median [range]. Statistical comparisons were made using the Mann-Whitney U test. CTRL, control; (T-)LCA, (tauro-)lithocholic acid; DCA, deoxycholic acid; CDCA, chenodeoxycholic acid; UDCA, ursodeoxycholic acid; HDCA, hyodeoxycholic acid; CA, cholic acid; MCA, muricholic acid. [a]Slight overestimation possible due to interfering peak.

Supplemental Table S2. Concentrations of individual bile acid species in plasma

Bile acid	CTRL (n=8)	*Cyp2c70*^{ako} (n=8)	p-value
LCA (μmol/L)	0.0 [0.0-0.0]	0.2 [0.1-0.4]	0.0002
DCA (μmol/L)	0.3 [0.1-0.4]	0.5 [0.3-1.9]	0.003
CDCA (μmol/L)	0.0 [0.0-0.1]	1.5 [0.2-4.7]	0.0002
UDCA (μmol/L)	0.1 [0.0-0.2]	0.7 [0.1-2.7]	0.0006
HDCA (μmol/L)	0.0 [0.0-0.0]	0.0 [0.0-0.1]	0.08
CA (μmol/L)	0.4 [0.1-2.4]	0.8 [0.3-6.6]	0.16
αMCA (μmol/L)	0.0 [0.0-0.1]	0.1 [0.0-0.4]	0.005
βMCA (μmol/L)	0.2 [0.1-2.2]	0.1 [0.0-0.2]	0.005
ωMCA[a] (μmol/L)	0.3 [0.1-2.1]	0.1 [0.0-0.3]	0.04
T-LCA (μmol/L)	0.0 [0.0-0.0]	0.1 [0.0-0.2]	0.0002
T-DCA (μmol/L)	0.1 [0.0-0.4]	0.5 [0.1-2.4]	0.02
T-CDCA (μmol/L)	0.0 [0.0-0.1]	2.9 [0.8-17.9]	0.0002
T-UDCA (μmol/L)	0.1 [0.1-0.2]	0.7 [0.1-4.2]	0.0003
T-HDCA (μmol/L)	0.0 [0.0-0.1]	0.0 [0.0-0.1]	0.43
T-CA (μmol/L)	2.5 [0.9-11.6]	4.0 [1.6-24.1]	0.33
T-αMCA (μmol/L)	0.2 [0.1-0.7]	1.0 [0.4-7.4]	0.001
T-βMCA (μmol/L)	0.9 [0.3-3.0]	0.2 [0.1-1.0]	0.02
T-ωMCA (μmol/L)	0.3 [0.2-0.9]	0.1 [0.0-0.4]	0.01
Total bile acids (μmol/L)	6.2 [2.1-18.1]	16.2 [5.3-74.9]	0.050

Bile acid species in plasma of male mice presented as median [range]. Statistical comparisons were made using the Mann-Whitney U test. CTRL, control; (T-)LCA, (tauro-)lithocholic acid; DCA, deoxycholic acid; CDCA, chenodeoxycholic acid; UDCA, ursodeoxycholic acid; HDCA, hyodeoxycholic acid; CA, cholic acid; MCA, muricholic acid. [a]Slight overestimation possible due to interfering peak.

Supplemental Table S3. Concentrations of individual bile acid species in bile

Bile acid	CTRL (n=8)	*Cyp2c70*^{ako} (n=6)	CTRL + PX (n=6)	*Cyp2c70*^{ako} + PX (n=7)
LCA (mmol/L)	ND	ND	ND	ND
DCA (mmol/L)	ND	ND	ND	ND
CDCA (mmol/L)	ND	ND	ND	ND
UDCA (mmol/L)	ND	ND	ND	ND
HDCA (mmol/L)	ND	ND	ND	ND
CA (mmol/L)	0.1 [0.0-0.2]	0.1 [0.0-0.2]	ND	ND
αMCA (mmol/L)	ND	ND	ND	0.0 [0.0-0.2]
βMCA (mmol/L)	0.0 [0.0-01]	ND	0.0 [0.0-0.1]	ND
ωMCA (mmol/L)	0.0 [0.0-0.1]	ND	0.0 [0.0-0.1]	0.0 [0.0-0.1]
T-LCA (mmol/L)	ND	0.1 [0.0-0.1]*	ND	ND$^{\#}$
T-DCA (mmol/L)	0.5 [0.4-0.8]	0.6 [0.3-0.9]	0.0 [0.0-0.1]$^{\#}$	ND$^{\#}$
T-CDCA (mmol/L)	0.2 [0.1-0.2]	6.4 [5.4-13.4]*	0.1 [0.1-0.2]$^{\#}$	2.7 [1.4-12.4]*$^{,\#}$
T-UDCA (mmol/L)	0.3 [0.2-0.4]	0.7 [0.3-1.3]*	0.3 [0.1-0.4]	1.1 [0.9-3.4]*$^{,\#}$
T-HDCA (mmol/L)	0.1 [0.1-0.2]	0.2 [0.1-0.3]	0.1 [0.0-0.1]$^{\#}$	0.1 [0.0-0.4]$^{\#}$
T-CA (mmol/L)	19.8 [13.3-25.5]	13.9 [9.7-17.8]*	1.5 [0.5-2.3]$^{\#}$	1.4 [1.1-3.4]$^{\#}$
T-αMCA (mmol/L)	0.9 [0.4-1.0]	3.6 [1.7-3.9]*	0.6 [0.3-0.8]$^{\#}$	3.8 [2.4-12.4]*
T-βMCA (mmol/L)	8.9 [5.6-15.9]	1.1 [0.9-1.8]*	9.8 [5.0-11.8]	2.3 [1.4-7.3]*$^{,\#}$
T-ωMCA (mmol/L)	2.0 [1.4-3.1]	0.3 [0.2-0.6]*	2.0 [1.2-3.5]	0.8 [0.4-2.0]*$^{,\#}$
Total bile acids (mmol/L)	34.6 [22.4-47.1]	27.2 [19.2-38.6]	15.2 [7.2-18.3]$^{\#}$	11.8 [9.9-45.2]$^{\#}$

Bile acid species in cannulated bile bile of male mice presented as median [range]. Statistical comparisons were made using the Kruskal-Wallis H test followed by Conover *post hoc* comparisons. CTRL, control; PX, PX20606; (T-)LCA, (tauro-)lithocholic acid; DCA, deoxycholic acid; CDCA, chenodeoxycholic acid; UDCA, ursodeoxycholic acid; HDCA, hyodeoxycholic acid; CA, cholic acid; MCA, muricholic acid; ND, not detected. *$p<0.05$ vs. WT controls receiving the same treatment. $^{\#}p<0.05$ vs. animals of the same genotype not receiving PX.

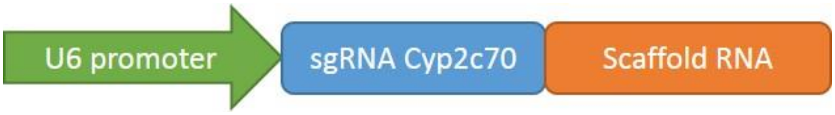

GAATTCGAGGGCCTATTTCCCATGATTCCTTCATATTTGCATATACGATACAAGGCTGTTAGAGAGATAATTGG
AATTAATTTGACTGTAAACACAAAGATATTAGTACAAAATACGTGACGTAGAAAGTAATAATTTCTTGGGTAGT
TTGCAGTTTTAAAATTATGTTTTAAAATGGACTATCATATGCTTACCGTAACTTGAAAGTATTTCGATTTCTTGGC
TTTATATATCTTGTGGAAAGGACGAAACACCGCCCACTCCTTTACCAATTGTGTTTTAGAGCTAGAAATAGCAA
GTTAAAATAAGGCTAGTCCGTTATCAACTTGAAAAAGTGGCACCGAGTCGGTGCTTTTTTGCGGCCGC

U6 promoter
Guide RNA sequence
Scaffold

Supplemental Figure S1. Single-guide RNA expression cassette used to target the Cyp2c70 gene

Nucleotide sequence of the expression cassette that was cloned into the adenovirus that was used to acutely knock-out Cyp2c70 in livers of L-Cas9tg mice. See methods section for details concerning the production of the virus and further experimental details.

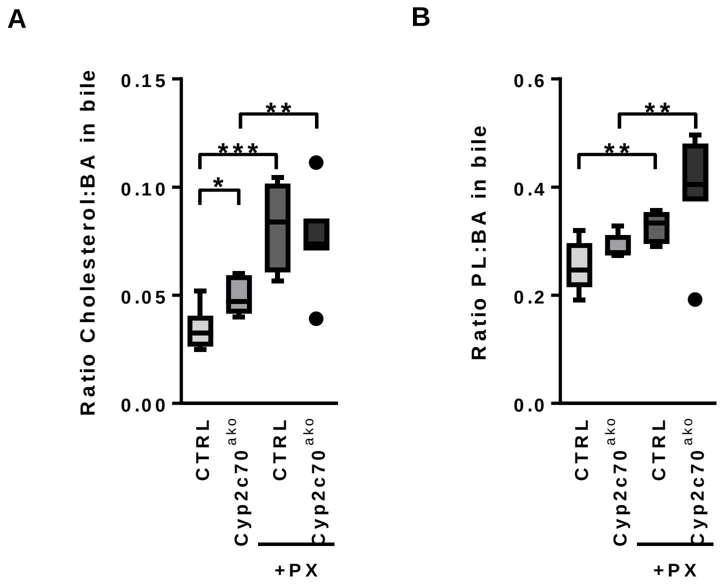

Supplemental Figure S2: Bile composition

Ratios of cholesterol:bile acids as well as phospholipids:bile acids in bile of *Cyp2c70*^{ako} mice and controls ±PX20606 (PX; 10mg/kg/day). *p<0.05, **p<0.01, ***p<0.001 (Kruskal-Wallis H test followed by Conover *post-hoc* comparisons). N=6-8 animals/group. BA, bile acids; CTRL, control; PL, phospholipids.

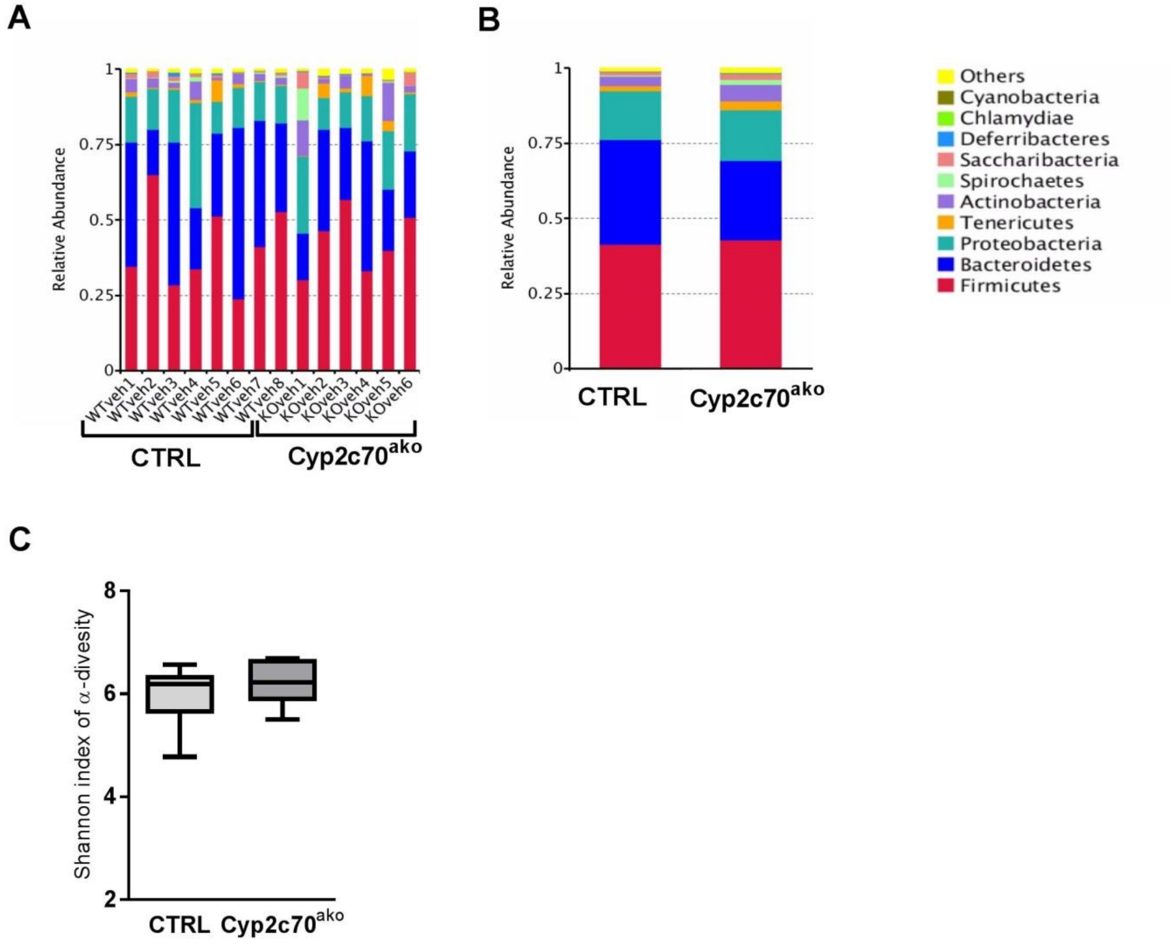

Supplemental Figure S3. Impact of *Cyp2c70* ablation on microbiota

Microbial DNA was extracted from freshly frozen feces and 16S ribosomal DNA was sequenced. Stack diagrams showing the relative abundance of different phyla in the individual mice (A) are provided as well as the mean of the groups (B). The Shannon index, providing information concerning the diversity of the microbiota did not reveal differences between the groups (C). N=6-8 animals/group.

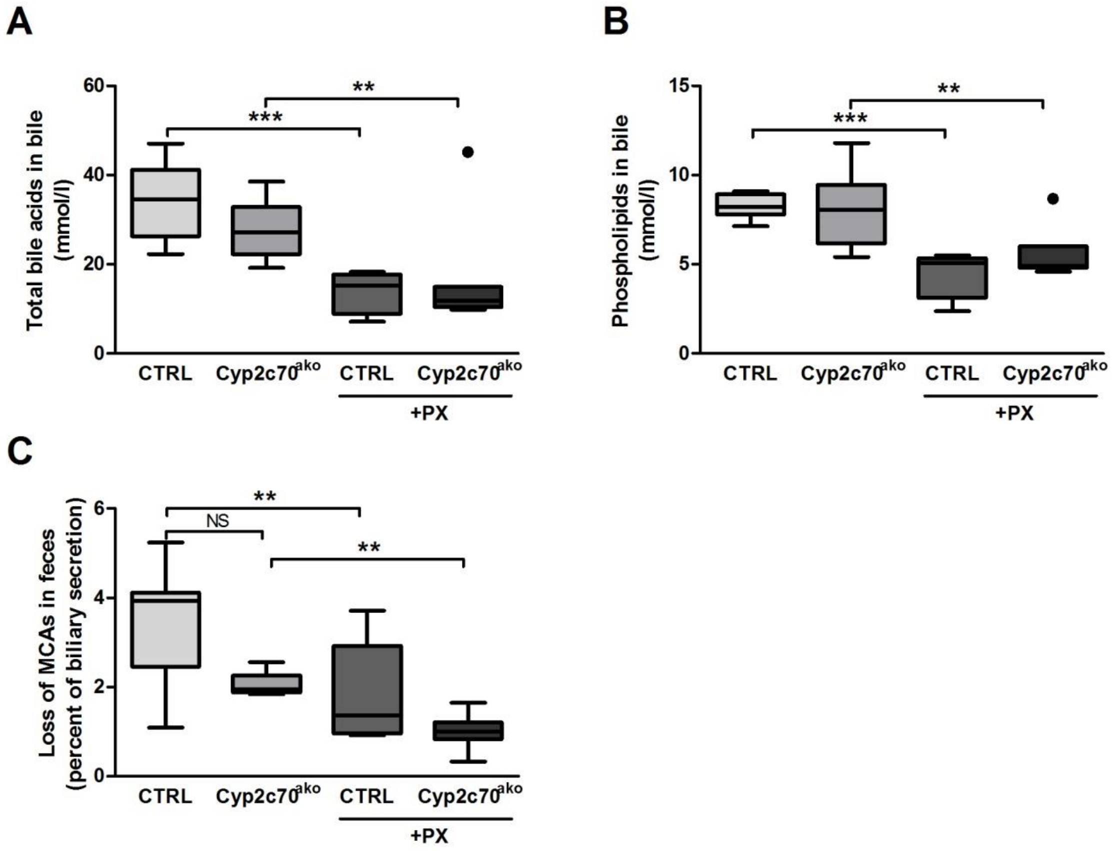

Supplemental Figure S4. Concentrations of bile acids and phospholipids in bile and fecal loss of muricholic acids

Biliary bile acid (A) and phospholipid (B) concentrations in *Cyp2c70*^{ako} mice and controls ±PX20606 (PX; 10mg/kg/day). Percentage of muricholic acids secreted in bile that is lost in feces (C), where lower percentages loss thus represent more efficient intestinal reabsorption. **$p<0.01$, ***$p<0.001$ (Kruskal-Wallis H test followed by Conover *post-hoc* comparisons). N=6-8 animals/group. CTRL, control; MCAs, muricholic acids; NS, not significant.

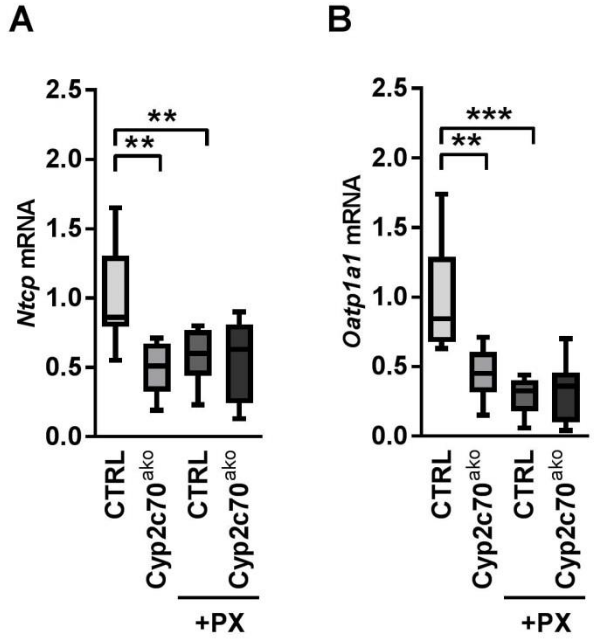

Supplemental Figure S5. Hepatic mRNA expression of bile acid uptake transporters

Expression of the bile uptake transporters *Ntcp* (A) and *Oatp1a1* (B) was determined by real-time quantitative PCR on reverse transcribed RNA. **p<0.01, ***p<0.001 (Kruskal-Wallis H test followed by Conover *post-hoc* comparisons). N=6-8 animals/group. CTRL, control; PX, PX20606.
